# Unraveling Patterns of Congenital Structural Malformations in Infants: A Hospital-Based Descriptive Study

**DOI:** 10.7759/cureus.60375

**Published:** 2024-05-15

**Authors:** Savita Pandey, Yasir A Lone, Saikat Patra, Braham P Kalra, Sanyam Modi

**Affiliations:** 1 Pediatrics, Himalayan Institute of Medical Sciences, Swami Rama Himalayan University, Dehradun, IND; 2 Pediatric Surgery, Himalayan Institute of Medical Sciences, Dehradun, IND; 3 Neonatology, Himalayan Institute of Medical Sciences, Dehradun, IND; 4 Pathology, Smt. B.K. Shah (SBKS) Medical Institute & Research Centre, Vadodara, IND

**Keywords:** external anomalies, internal anomalies, congenital structural anomalies, congenital anomalies, congenital malformations

## Abstract

Introduction

Congenital malformation studies serve several purposes, including establishing baseline rates, monitoring changes over time, exploring the origins of these defects, and helping in planning health services. Increasing public awareness about pediatric surgical interventions is another goal of these studies. However, the impact of congenital malformations is often underestimated in developing countries due to insufficient healthcare data and diagnostic facilities, particularly in rural areas. Families affected by the birth of a child with congenital malformations face significant stress and hardship.

Methods

The main aims of this study were to evaluate the clinical pattern of congenital structural malformations in our region (Uttarakhand, India), identify possibly associated factors of congenital malformations, and find out the immediate outcome of congenital malformations in enrolled participants.

Results

Among a total of 150 cases, 73 (48.7%) cases were inborn, whereas 77 (51.3%) cases were outborn. Investigation of congenital malformation revealed cleft lip or palate in 37 (24.7%) cases, congenital heart disease (CHD) in 33 (22%) cases, meningomyelocele (MMC) in 18 (12.0%) cases, anorectal malformation (ARM) in 11 (7.3%) cases, hypospadias in 10 (6.7%) cases, congenital talipes equinovarus (CTEV) in nine (6.0%) cases, tracheoesophageal fistula (TEF) in nine (6.0%) cases, polydactyly in seven (4.7%) cases, pelviureteric junction obstruction (PUJO) in four (2.7%) cases, duodenal atresia in three (2.0%) cases, midgut volvulus in three (2.0%) cases, umbilical sinus in two (1.3%) cases, sacrococcygeal teratoma (SCT) in one (0.7%) case, phimosis in one (0.7%) case, microtia in one (0.7%) case, and micrognathia in one (0.7%) case. Mortality was observed in 11 (7.3%) cases, whereas 105 (70%) cases were successfully discharged. Among 11 mortality cases, the cause of death was CHD in seven (63.2%) cases, TEF+CHD in two (18.1%) cases, MMC in one (9%) case, and duodenal atresia in one (9%) case.

Conclusion

Contrary to the common belief that advanced maternal age of greater than 35 years is a major cause, 86.6% of the congenital structural anomalies in our hospital-based study in Uttarakhand occurred in babies of mothers belonging to the age group of 18-30 years. Also, consanguineous marriage was observed in only 3.3% of cases, indicating that it may not be a major contributing factor causing congenital structural malformations in our region. External congenital anomalies are most commonly observed (60.7%), with cleft lip and cleft palate being the most common. The most frequently observed internal congenital anomaly is CHD (22%) followed by gastrointestinal (GI) (18.6%) and urinary anomalies (10.1%). Death and referral are commonly seen in CHD.

## Introduction

Congenital malformations, also known as birth defects, encompass abnormalities in newborn organ systems that disrupt shape and function, occurring during intrauterine life and detected before, around, or after birth [1]. The World Health Organization (WHO) originally defined congenital malformations as only structural birth abnormalities in 1972, but the definition was expanded in 2012 to include metabolic diseases present at birth [2]. These defects significantly increase neonatal and infant mortality and morbidity, posing a serious public health concern.

Various factors contribute to the etiology of birth defects, including genetic disorders, inadequate nutrition, alcohol consumption, exposure to environmental pollutants such as pesticides and tobacco smoke, maternal venereal diseases, and advanced maternal age [3]. Chromosomal abnormalities, exposure to teratogens, genetics combined with environmental factors, and single gene disorders are among the suggested causes [3]. Notably, around 50% of congenital malformations have no identified cause [3].

Congenital abnormalities significantly contribute to newborn mortality in both industrialized and developing nations, with global prevalence ranging from 2% to 3% [1]. Regional variations exist. They are a leading cause of miscarriage and have adverse effects on mothers, families, and child health, increasing the risk of premature delivery and causing morbidity in childhood and adulthood. Worldwide, birth defects cause approximately 240,000 infant deaths within 28 days after birth annually, with an additional 170,000 deaths in children aged one month to five years. Ninety percent of severe birth defects occur in low- and middle-income countries, with heart problems, neural tube defects, and Down's syndrome being the most common [4]. In India, approximately 1.7 million babies are born with birth defects annually, contributing to 8%-15% of prenatal mortality and 13%-16% of newborn deaths [5].

Congenital malformations can affect multiple organ systems depending on the timing of damage during embryogenesis. Central nervous system anomalies are cited as the most common in some studies, while digestive system abnormalities are identified as the most frequent in others [1]. "Dysplasia" refers to irregular cell arrangement within tissues, while "deformation" refers to changes in shape or structure caused by external forces unless stemming from a persistent issue like a bicornuate uterus, the recurrence risk for deformation is usually low [6]. "Disruption" denotes the destruction of a normally developed structure due to an insult. A "syndrome" comprises multiple anomalies with a common pathophysiological cause and a defined etiology. "Sequences" involve deformities resulting from a single incident with diverse etiologies, while an "association" refers to a non-random grouping of malformations lacking a clear link among them [7].

Improving the detection of congenital abnormalities is best achieved through fetal anomaly scanning. In cases of significant uncorrectable anomalies, termination of pregnancy can be considered. Alternatively, if a correctable defect is detected, delivery should occur in a center equipped with pediatric surgical and neonatal intensive care facilities [8]. Without proper care, survivors of congenital abnormalities may experience lifelong physical, mental, visual, or auditory challenges, significantly impacting not only their social and professional lives but also those of their families and communities. Parents of newborns with significant defects often experience emotional reactions such as denial, guilt, sadness, and shame, even if the abnormalities were detected prenatally, highlighting the importance of appropriate counseling [9].

It is crucial to create population-based registries for the epidemiological surveillance of congenital malformations to standardize records and offer more precise data regarding the prevalence of common and uncommon anomaly types. The International Clearinghouse for Birth Defects Surveillance and Research is a volunteer non-profit global organization, which is officially affiliated with the WHO. It brings together global research and monitoring initiatives for congenital malformations to study, prevent, and lessen the impact of these conditions. It does not function in many regions of developing countries, nor do any other worldwide surveys on birth abnormalities [10].

Congenital malformation studies serve several purposes, including establishing baseline rates, monitoring changes over time, and providing insights into the origins of these defects. They also help in planning health services, especially in high-risk populations undergoing prenatal screening. Increasing public awareness about pediatric surgical interventions is another goal of these studies. However, the impact of congenital malformations is often underestimated in developing countries due to insufficient healthcare data and diagnostic facilities, particularly in rural areas. Therefore, implementing preventive and diagnostic measures to identify congenital abnormalities in infants is essential for addressing this issue [11].

Limited data exists regarding the frequency and distribution of congenital malformations in our area, highlighting the need to assess the occurrence, characteristics, and outcomes of these deformities in infants within our community. This study aims to address the information gap regarding the prevalence and distribution of congenital malformations in the local newborn population, eventually leading to the enhancement of healthcare planning.

## Materials and methods

The main aims of this study were to evaluate the clinical pattern of congenital structural malformations, to identify possible associated factors, and to find out the immediate outcome of congenital malformations in enrolled participants. The study was conducted under the Departments of Pediatrics, Pediatric Surgery, and Neonatology at our tertiary care teaching hospital for a period of 12 months (July 2022 to June 2023). The subjects of the study are neonates born at our hospital (inborn) or outside and infants up to one year of age admitted in the neonatal intensive care unit (NICU), postnatal ward, pediatric ward, pediatric surgery ward, and pediatric intensive care unit (PICU). The researcher conducted the study using a proforma with the patient attendant/representative. Written informed consent was taken from the guardians of all the subjects before enrolment in the study after clearance from the institutional ethical committee. This is a prospective, single-center, observational, descriptive, and hospital-based study.

All neonates and infants up to one year of age with congenital structural malformations were included in the study. Parents who did not give consent and patients in whom corrective procedures had already been carried out somewhere else were excluded from the study. Also, babies with incidentally diagnosed internal anomalies were also excluded. So, asymptomatic babies may have had some internal anomalies that might not have been picked up. For example, babies with asymptomatic horseshoe kidneys may have been missed. This becomes a limitation, but such asymptomatic internal anomalies were excluded from the study even if incidentally picked up. Stillbirths were also excluded.

After approval of the institutional ethics committee, the nature and purpose of the study were explained, and informed consent was obtained from the guardians of the children. Data was collected for the demography of children including age, gender, body weight, malnutrition, socioeconomic status, ethnicity, and place of residence. Detailed information regarding congenital malformations was obtained and recorded in the case reporting form. This study has been conducted as per National Ethical Guidelines for Biomedical and Health Research involving human participants (Indian Council of Medical Research [ICMR] 2017) guidelines.

The data was collected and entered in MS Excel 2010 (Microsoft Corp., Redmond, Washington), and statistical analysis was performed by using SPSS software version 27.0 (IBM Corp., Armonk, NY). Qualitative variables were represented in the form of frequency and percentage, while quantitative variables shall be expressed as mean ± SD. Any association between categorical variables was calculated by the Chi-square test or Fisher's exact test wherever applicable. Difference in the means was assessed by the student’s t-test and analysis of variance (ANOVA) test wherever applicable with suitable post-hoc analysis. A p-value < 0.05 was considered significant.

## Results

**Figure 1:**
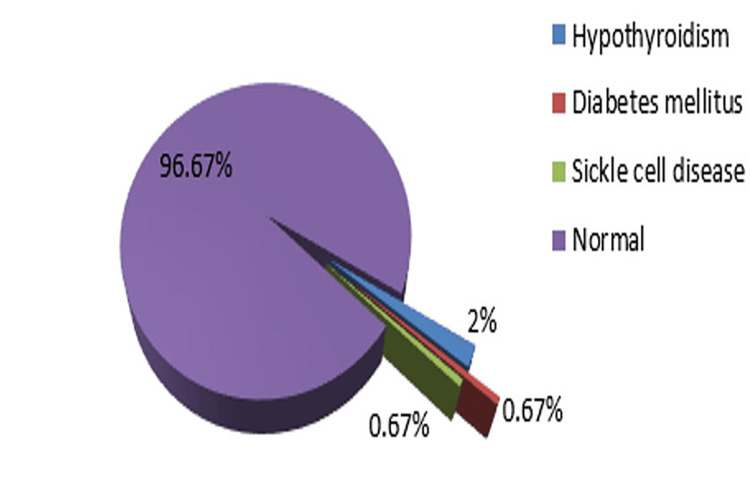
Associated chronic systemic diseases during pregnancy

The mean age of the mothers was 25.45 ± 4.12 years. Among a total of 150 women, 77 (51.3%) belong to the age group of 18-25 years, 53 (35.3%) belong to the age group of 26-30 years, 15 (10%) belong to the age group of 31-35 years, and five (3.3%) belong to the age group of 36-40 years. Among a total of 150 women, 75 (50%) were revealed to be primigravida, and 75 (50%) were found to be multigravida. Single gestation was observed in 145 (96.7%) cases, whereas multiple gestation occurred in five (3.3%) cases. Assessment of chronic systemic disease indicates the presence of hypothyroidism in three (2%) cases, diabetes in one (0.7%) case, and sickle cell anemia in one (0.7%) case (Figure 1).

**Figure 2:**
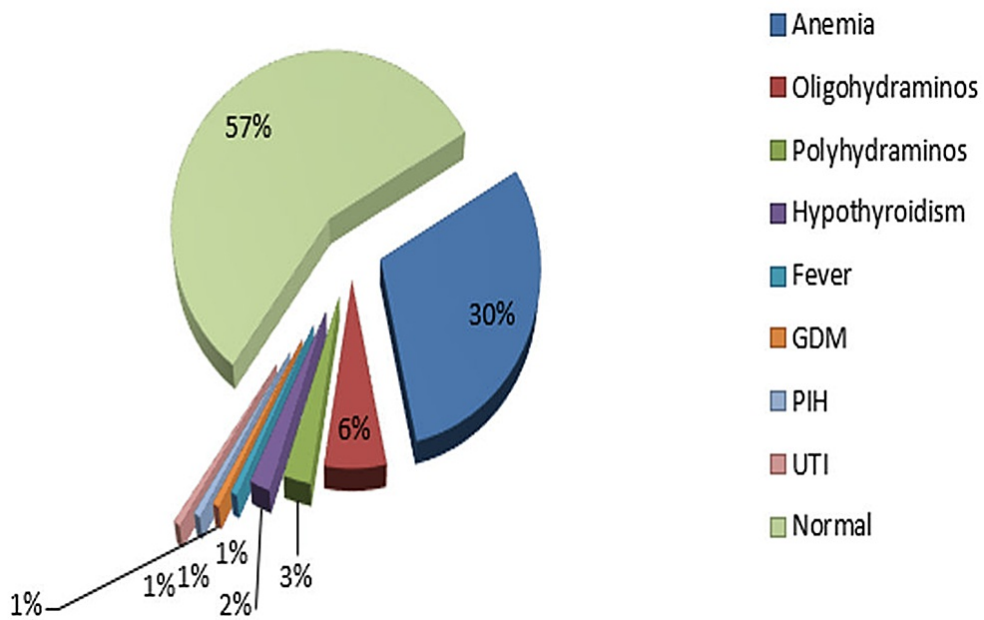
Assessment of problems occurring during pregnancy GDM: Gestational diabetes mellitus; PIH: Pregnancy-induced hypertension; UTI: Urinary tract infection.

Maternal nutrition status indicates optimum nutrition in 147 (98%) cases and malnutrition in three (2%) cases. Consanguineous marriage was observed in five (3.3%) cases. Assessment of problems that occurred during the pregnancy revealed anemia in 45 (30.0%) cases, oligohydramnios in nine (6.0%) cases, polyhydramnios in four (2.7%) cases, hypothyroidism in three (2.0%) cases, fever in one (0.7%) case, gestational diabetes in one (0.7%) case, pregnancy-induced hypertension (PIH) in one (0.7%) case, and urinary tract infection (UTI) in one (0.7%) case (Figure 2).

**Figure 3:**
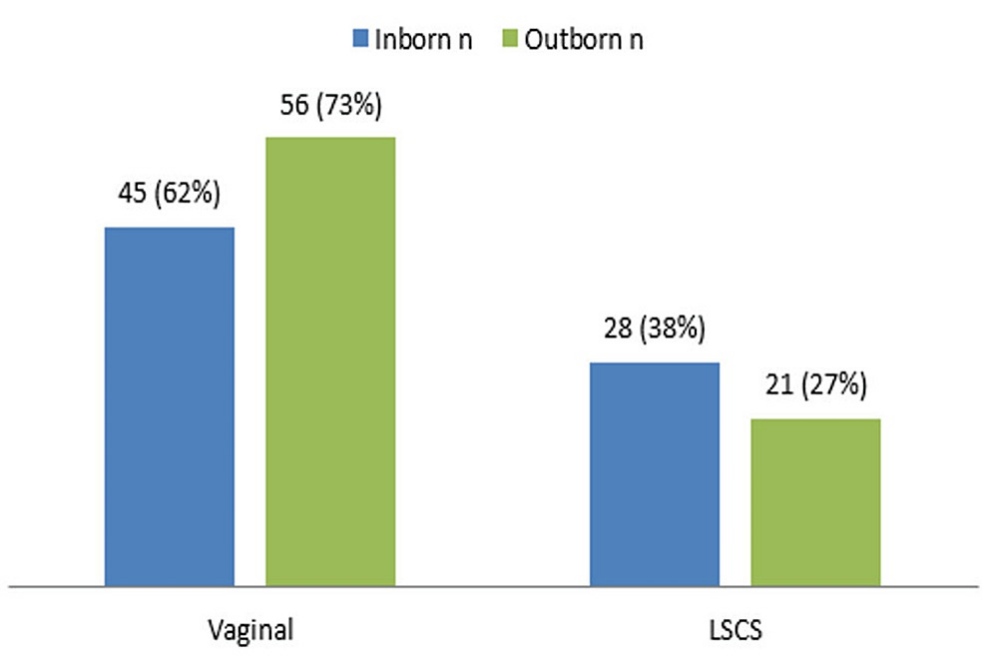
Mode of delivery LSCS: Lower segment cesarean section.

Gestation maturity indicates preterm birth in 26 (17.3%) cases, term birth in 122 (81.3%) cases, and post-term birth in two (1.3%) cases. Among 150 cases, 73 (48.7%) cases were inborn, whereas 77 (51.3%) cases were outborn. In the 73 inborn cases, vaginal mode of delivery was observed in 45 (61.6%) cases, whereas lower segment cesarean section (LSCS) was done in 28 (38.4%) cases. In the 77 inborn cases, vaginal mode of delivery was observed in 56 (72.7%) cases, whereas LSCS was done in 21 (27.3%) cases. There was no significant difference between the inborn and outborn cases in terms of mode of delivery (Figure 3).

**Figure 4:**
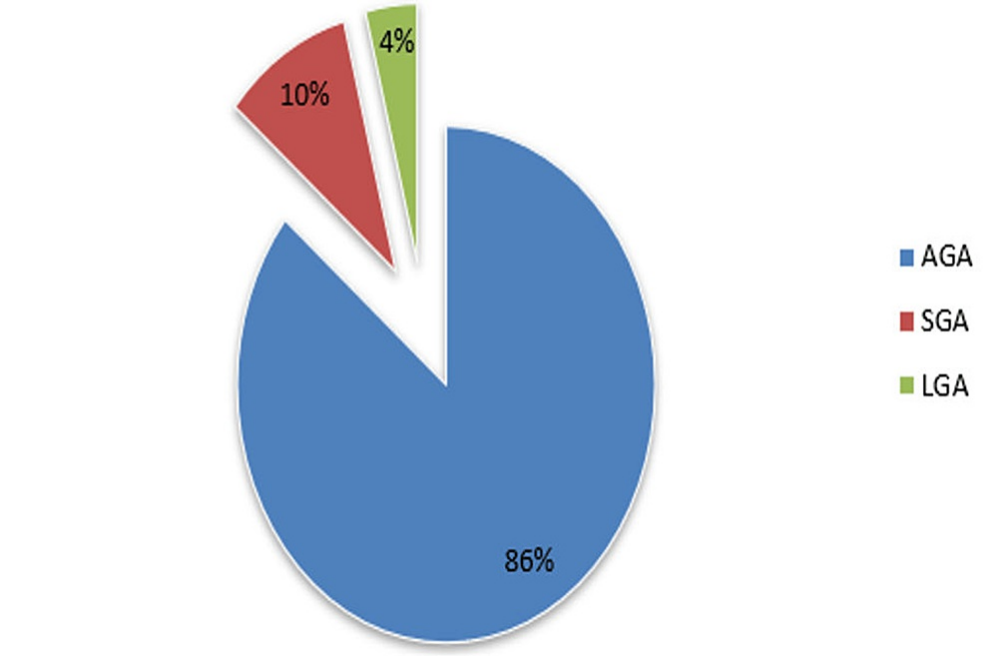
Birth weight as per gestational age AGA: Appropriate for gestational age; SGA: Small for gestational age; LGA: Large for gestational age.

Of the 73 inborn cases, 55 (75.3%) cases were male, and the rest of 18 (24.7%) cases were female. Of the 77 inborn cases, 49 (63.6%) cases were male, and the rest of 28 (36.4%) cases were female. There was no significant difference between the inborn and outborn cases in terms of the gender of the child. The mean birth weight was 2.50 ± 0.53 kg. Among a total of 150 cases, birth weight was <1 kg in one (0.7%) case, 1-1.49 kg in 10 (6.7%) cases, 1.5-2.49 kg in 44 (29.3%) cases, and 2.50-3.99 kg in 95 (63.3%) cases. Birth weight as per gestation age indicates appropriate for gestational age (AGA) in 129 (86.0%) cases, small for gestational age (SGA) in 15 (10.0%) cases, and large for gestational age (LGA) in six (4.0%) cases (Figure 4).

**Figure 5:**
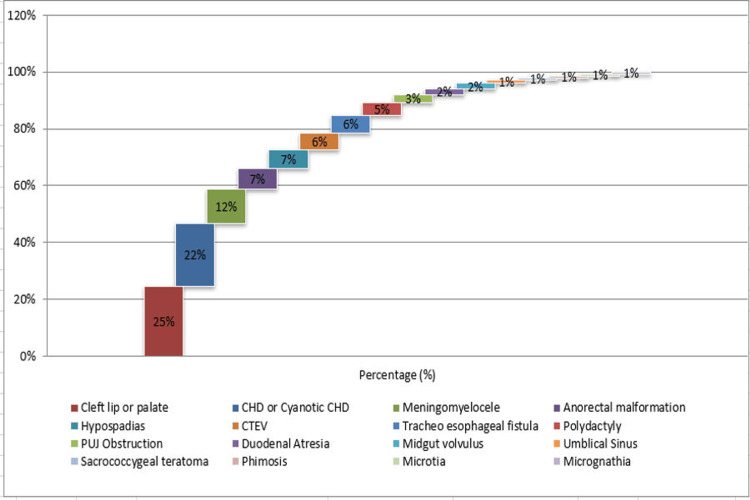
Assessment of congenital malformations CHD: Congenital heart disease; CTEV: Congenital talipes equinovarus; PUG: Pelviureteric junction.

Assessment of exposures to teratogens before/after pregnancy indicates exposure to tobacco in two (1.3%) cases and levetiracetam drug in one (0.7%) case. Analysis of associated comorbidities indicates hydrocephalus in four (2.7%) cases; undescended testis in three (2.0%) cases; CHD in two (1.3%) cases; pneumonia in one (0.7%) case; Down syndrome in one (0.7%) case; microtia, underdeveloped thumb, and micro-penis in one (0.7%) case; polydactyl in one (0.7%) case; and scrotum bifid, micro-penis, and left ectopic kidney in two (1.3%) cases. Antiepileptic drugs such as levetiracetam and phenobarbitone were reported in four (2.7%) cases. Investigation of congenital malformation revealed cleft lip or palate in 37 (24.7%) cases, CHD or cyanotic CHD in 33 (22%) cases, meningomyelocele in 18 (12.0%) cases, anorectal malformation in 11 (7.3%) cases, hypospadias in 10 (6.7%) cases, CTEV in nine (6.0%) cases, tracheoesophageal fistula in nine (6.0%) cases, polydactyly in seven (4.7%) cases, PUJ obstruction in four (2.7%) cases, duodenal atresia in three (2.0%) cases, midgut volvulus in three (2.0%) cases, umbilical sinus in two (1.3%) cases, sacrococcygeal teratoma in one (0.7%) case, phimosis in one (0.7%) case, microtia in one (0.7%) case, and micrognathia in one (0.7%) case (Figure 5).

**Figure 6:**
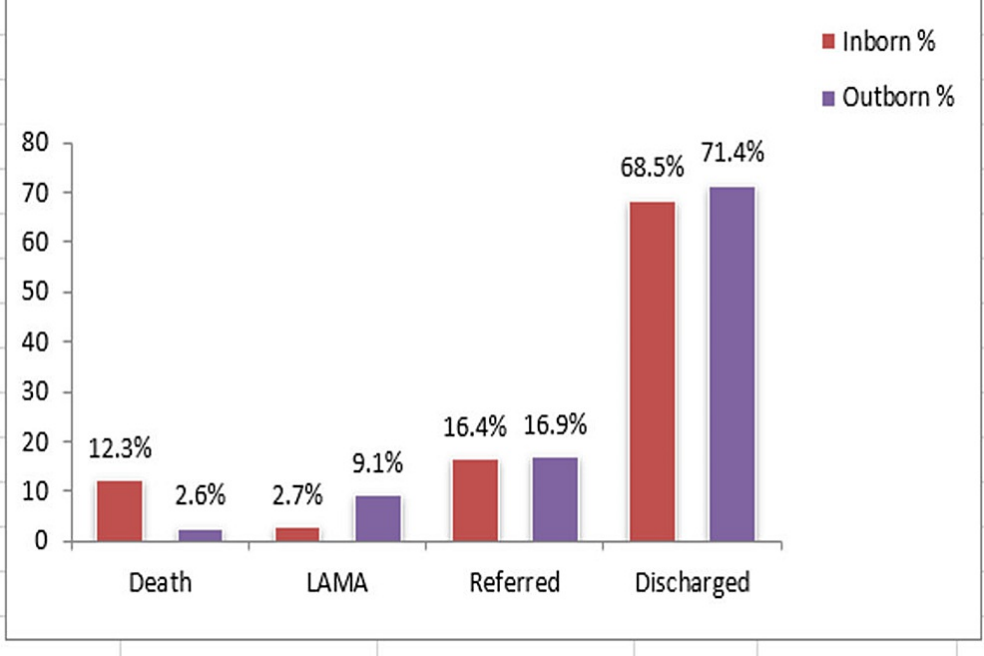
Outcome analysis LAMA: Leaving against medical advice.

External birth defect was reported in 91 (60.7%) cases, and internal birth defects were reported in 47 (31.3%) cases, whereas both defects were encountered in 12 (8%) cases. Major anomalies were observed in 124 (82.7%) cases, and minor anomalies were observed in 22 (14.7%) cases, whereas both kinds of anomalies were reported in four (2.7%) cases. Mortality was observed in 11 (7.3%) cases, whereas 105 (70%) cases were successfully discharged. Nine (6%) cases have taken the LAMA (leaving against medical advice), and 25 (16.6) cases were referred to specialized medical institutes (Figure 6).

**Figure 7:**
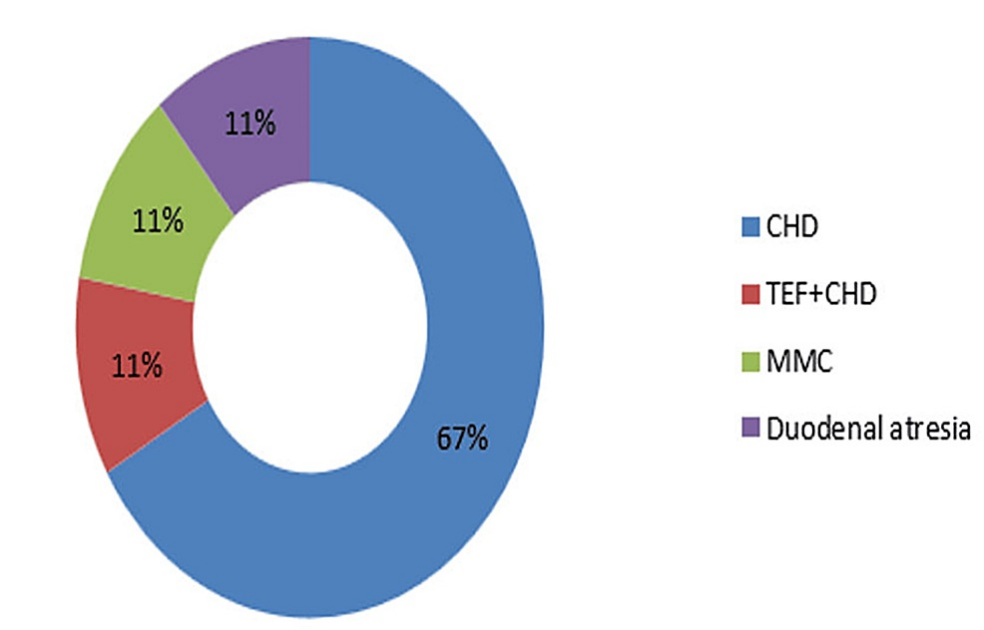
Causes of death CHD: Congenital heart disease; TEF: Tracheoesophageal fistula; MMC: Meningomyelocele.

Among nine mortality cases, the cause of death was CHD in six (67%) cases, TEF+CHD in one (11%) case, MMC in one (11%) case, and duodenal atresia in one (11%) case (Figure 7).

## Discussion

Recognizing the significance of birth abnormalities, the Indian government's Ministry of Health and Family Welfare launched the Rashtriya Bal Swasthya Karyakram (RBSK) initiative to address the issue effectively. At every delivery site, a thorough newborn screening program has been put in place as part of this initiative, with an emphasis on apparent birth abnormalities [12]. Congenital anomaly patterns and prevalence can change over time or depending on geographic region. It is influenced by genetic, environmental, and sociocultural influences, as well as racial and ethnic characteristics. In developing nations like India, congenital abnormalities are now major contributors to perinatal mortality as mortality due to other causes has been reduced due to better management of infections and nutritional deficiency illnesses [13].

The advanced age of the mother has been associated with a high rate of structural congenital malformation. Research by Seba et al. and Ghosh et al. [13,14] indicated that congenital malformations are commonly observed in mothers over the age of 30, while our study showed that most mothers of neonates with congenital anomalies were between 18 and 30 years of age. Our results are consistent with the study by Sanchez-Nadales et al., which showed that most mothers of neonates with congenital anomalies were between 20 and 35 years of age [12]. Similar findings were reported by Tiwari and Gupta [15]. Suguna et al. reported a higher incidence of malformation in babies born to mothers aged over 35 years [16]. Dutta et al. documented a statistically insignificant association between increased maternal age and congenital anomalies [17].

Consanguineous marriage was also reported to be linked with high rates of congenital malformation in the study by Seba et al. [13]. Other studies by Al-Gazali et al. [18], Ambe et al. [19], and Hollier et al. [20] also reported that consanguineous marriages play a major role in the occurrence of congenital malformations [19-21]. In the study by Bhalerao and Bhalerao, the prevalence of malformed babies was more when born out of consanguineous marriages [22]. In the study by Fadnis and Shankuwar, consanguinity was seen in five mothers whose babies had congenital anomalies (20%) [21]. Agarwal et al. [23] and Desai et al. [24] found a highly significant correlation between congenital malformation and consanguinity. AbouEl-Ella et al. study history of consanguinity was positive in 43% of couples [25]. An Egyptian study also reported that consanguinity was significantly associated with the presence of congenital anomalies (3.67%) compared with control populations (1.15%) [26]. However, in the present study, consanguineous marriage was observed in five (3.3%) cases.

The risk of congenital anomalies (excluding terminations) for gestational diabetes is 1.2 times higher than that in the total population [27]. In our study, anemia is the most common complication during pregnancy in 30% of cases, and gestational diabetes and case PIH were present in 0.7% of cases. The results of the present study are corroborated by the findings of Jayasree and D’Couth who observed that congenital anomalies are significantly associated with anemia and diabetes [28]. Similar findings were also obtained in the study of Padmanabhan et al. [29].

Studies have shown that congenital malformations are more common in males as compared to females. Similar observations have been reported in a present study in which 75.3% of inborn males and 63.6% of outborns were found to have congenital malformations. A study by Sanchez-Nadales et al. observed a higher prevalence of congenital malformations in male subjects [12]. In the study by Seba et al., 2.7% of male neonates were found to have structural congenital anomalies as compared to female children with a prevalence of 1.9% [13]. In a study done by Padmanabhan et al., male babies were more affected with malformations. About 54% of malformed babies were male and 45% were female babies [29]. A study by Taksande et al. showed similar results (61% of male babies and 37.4% of female babies) [30]. The high prevalence of congenital malformations in males could be because the female babies were affected by more lethal congenital malformations; so, they could not survive being born with signs of life [22]. In our present study, a higher birth weight was associated with a higher rate of congenital malformations, which is consistent with the findings from Sanchez-Nadales et al. [12] and Singh et al. [9]. However, this contradicts the findings of Seba et al. [13] who reported a high rate of congenital malformations in fetuses with lower birth weights.

Studies conducted by Seba et al. [13] and Bhalerao and Bhalerao [22] identified anomalies in the musculoskeletal system as the most prevalent form of congenital malformations. According to the WHO's fact sheet on congenital disorders, congenital heart defects are the most common form of severe congenital malformations, which is consistent with our study findings showing cleft lip or palate and congenital heart defects as the most common congenital anomalies.

In our study, a mortality rate of 7.3% was observed among cases of congenital malformations, which was consistent with the findings from Sanchez-Nadales et al. [12] who reported a mortality rate of 11.5%. Variations in mortality rates across different regions may be attributed to social and racial influences commonly observed in genetic disorders. Additionally, factors such as the background of investigators, sample selection, and study period can contribute to varying results.

The congenital anomaly prevalence in our hospital-based research may surpass general population rates due to sampling limitations. Focused solely on hospital cases, our study did not aim for community representation. Community-based research could yield higher rates, capturing cases like abortions and stillbirths not seen in hospitals. Tertiary care hospitals lack defined catchment areas, complicating prevalence generalization. Accurate estimation requires community-based research. In developing nations like India, approved preventive measures are lacking despite high recurrence likelihood. Awareness of prenatal care benefits and implementation of sensitive screening tests are crucial to reduce both incidence and impact.

## Conclusions

Contrary to the common belief of advanced maternal age of greater than 35 years being a major cause, 86.6% of the congenital structural anomalies in our hospital-based study in Uttarakhand occurred in babies of mothers belonging to the age group of 18-30 years. Also, consanguineous marriage was observed in only 3.3% of cases, indicating that it may not be a major contributing factor causing congenital structural malformations in our region.

External congenital anomalies are most commonly observed (60.7%) followed by internal and external anomalies. Among external congenital anomalies, cleft lip and cleft palate are most common. The most commonly observed internal congenital anomaly is CHD (22%) followed by GI anomalies (18.6%) and urinary anomalies (10.1%). Death and referral are commonly seen in CHD either due to uncorrectable nature or due to the unavailability of a neonatal cardiac thoracic vascular surgeon. No obvious associated factor was observed in our study.
